# Understanding Monitoring Technologies for Adults With Pain: Systematic Literature Review

**DOI:** 10.2196/jmir.7279

**Published:** 2017-10-27

**Authors:** Iyubanit Rodríguez, Valeria Herskovic, Carmen Gerea, Carolina Fuentes, Pedro O Rossel, Maíra Marques, Mauricio Campos

**Affiliations:** ^1^ Department of Computer Science Pontificia Universidad Católica de Chile Santiago Chile; ^2^ School of Computer Science University of Nottingham Nottingham United Kingdom; ^3^ Department of Computer Science Universidad Católica de la Santísima Concepción Concepción Chile; ^4^ Department of Computer Science Universidad de Chile Santiago Chile; ^5^ Faculty of Medicine Pontificia Universidad Católica de Chile Santiago Chile

**Keywords:** systematic review, pain, technology, patient monitoring, ubiquitous and mobile computing

## Abstract

**Background:**

Monitoring of patients may decrease treatment costs and improve quality of care. Pain is the most common health problem that people seek help for in hospitals. Therefore, monitoring patients with pain may have significant impact in improving treatment. Several studies have studied factors affecting pain; however, no previous study has reviewed the contextual information that a monitoring system may capture to characterize a patient’s situation.

**Objective:**

The objective of this study was to conduct a systematic review to (1) determine what types of technologies have been used to monitor adults with pain, and (2) construct a model of the context information that may be used to implement apps and devices aimed at monitoring adults with pain.

**Methods:**

A literature search (2005-2015) was conducted in electronic databases pertaining to medical and computer science literature (PubMed, Science Direct, ACM Digital Library, and IEEE Xplore) using a defined search string. Article selection was done through a process of removing duplicates, analyzing title and abstract, and then reviewing the full text of the article.

**Results:**

In the final analysis, 87 articles were included and 53 of them (61%) used technologies to collect contextual information. A total of 49 types of context information were found and a five-dimension (activity, identity, wellness, environment, physiological) model of context information to monitor adults with pain was proposed, expanding on a previous model. Most technological interfaces for pain monitoring were wearable, possibly because they can be used in more realistic contexts. Few studies focused on older adults, creating a relevant avenue of research on how to create devices for users that may have impaired cognitive skills or low digital literacy.

**Conclusions:**

The design of monitoring devices and interfaces for adults with pain must deal with the challenge of selecting relevant contextual information to understand the user’s situation, and not overburdening or inconveniencing users with information requests. A model of contextual information may be used by researchers to choose possible contextual information that may be monitored during studies on adults with pain.

## Introduction

Monitoring involves repeated study of a question that requires collecting data [1] in real time [2]. Patient monitoring technology aims to manage, control, and treat patients while collecting information from their environment [3]. The number of health monitoring apps has increased in recent years because they may reduce health care costs [4]. Since monitoring is done in the patient’s environment, it is necessary to understand information about their situation or context. Context may be defined as “any information that can be used to characterize the situation of entities (ie, whether a person, place, or object) that are considered relevant to the interaction between a user and an application, including the user and the application themselves” [5]. The aim of context-aware computing is “to acquire and use data about the context of a device to provide services that are appropriate for the particular setting” [6]. For example, sensors may be used to gather contextual information, such as trunk posture [7,8], and provide feedback so users can improve their posture [9,10].

Pain is an “unpleasant sensory and emotional experience associated with actual or potential tissue damage” [11]. Pain is the most common diagnosis and problem that patients seek help for in hospitals [12]. Pain assessment is done primarily through subjective reports of patients, caregivers, and medical staff, but these reports have several limitations (eg, inconsistent metrics, reactivity to suggestions, and that they cannot be used with children or patients with certain neurological impairments) [13,14]. Additionally, pain is usually evaluated during a medical appointment [15], which means the physician does not have information about how the patient feels during his/her daily routine or how other factors may affect pain intensity. Therefore, patients may benefit from being monitored, since physicians may acquire a more complete and realistic assessment of the patient’s situation. There is a large amount of possible contextual information that may be captured, so which data are relevant will depend on the particular situation being studied.

The aim of this work is to determine what types of technologies have been used to monitor adults with pain and propose a model of context information relevant to patients with pain. For this, a systematic literature review (SLR) was conducted, which is a means to identify, evaluate, and interpret all relevant research available for a research question or topic [16].

## Methods

A SLR was conducted following Kitchenham and Charter’s guidelines for performing SLRs [16]. The review protocol describes all steps performed during the review, reduces risk of bias, and increases its rigor, transparency, and repeatability [17].

### Search Strategy

A systematic search of published literature was conducted to analyze recent research about context information related to pain and technologies used to monitor adults with pain. The search was conducted electronically during October 2015 in the following digital libraries: ACM Digital Library, IEEE Xplore Digital Library, ScienceDirect, and PubMed. These libraries were chosen to cover medical and technological aspects. This review was limited to articles published between 2005 and 2015, and duplicate citations across databases were identified and excluded using the Papers software.

The keywords were identified by consulting with medical specialists on appropriate words, manually selecting publications related to the subject, and analyzing frequently used words. The set of keywords was (context-sensitive, context-aware, physiological, environment*) AND (monitor*, sens*, measure*) AND (pain). The asterisk operator (*) indicates that there may be more letters after the root word. With these keywords, the search string was built using Boolean AND and OR operators. The search string was input into each database and the keywords were restricted to be found in the abstract and/or document title and published on or after January 1, 2005. In total, 1758 articles were retrieved, with the following distribution according to the consulted database: ACM (n=113), IEEE (n=55), ScienceDirect (n=548), and PubMed (n=1042).

It is relevant to note that other keywords were tested in the search engines, most notably the word “context.” However, a large number of articles use “context” as the context of the study itself, so the words “context sensitive” and “context aware” were used instead.

### Selection Criteria

A study was included in this review if it met the following inclusion criteria: (1) it presented a study of context information and pain; (2) the study was carried out on adults; (3) the article was peer reviewed and it was obtained from a journal, conference, or workshop; (4) it was published between January 1, 2005 and October 1, 2015; and (5) the study was published in English. Articles were excluded if they presented studies pertaining to animals, plants, robots, or children, or if the study was a literature review, mapping study, SLR, only presented as an abstract, or if it was not possible for any of the authors to download the full text of the article (no access through university subscriptions).

### Selection Process

The included articles were selected through two steps. In the first step, title, publication venue, year of publication, and abstract for each article were collected in an Excel spreadsheet. Two reviewers assessed each publication (IR reviewed all articles; MM and PR each reviewed half) and applied the inclusion/exclusion criteria. Publications with two votes to include or exclude were automatically included or excluded. Publications with differing votes were sent to a third reviewer (VH), who analyzed it and determined whether the publication should be included or not.

In the second step, the primary and secondary reviewers (IR, CG) read the full text of a random sample of 10 publications. Each reviewer independently assessed whether the article should be accepted or rejected. Then, Cohen kappa was calculated, with a result of 1, which suggested that the inclusion/exclusion criteria were clear enough to be applied consistently [18]. Each reviewer also filled out a table of questions in Excel composed of 29 criteria, which were then discussed to clarify the questions and rewrite them if necessary.

Finally, an accelerated liberal approach was applied [19], in which the first reviewer (IR) read the full text of all the publications and rejected those that did not meet the inclusion/exclusion criteria (corresponding to 27 articles). CG validated the rejected publications. There were eight disagreements, which were solved by a third reviewer (CF), who analyzed them and determined whether the publication should be accepted.

### Data Extraction and Quality Assessment

One reviewer (IR) extracted information from each publication using a predesigned Excel spreadsheet with 29 columns (eg, authors; study date; study purpose; country; contextual information; activity being monitored; main user; number of participants; study methodology, such as methods used, number and type of participants, activity; type of monitoring technology used). Quality of studies was not considered in this analysis.

## Results

### Selection and Inclusion of Studies

**Figure 1:**
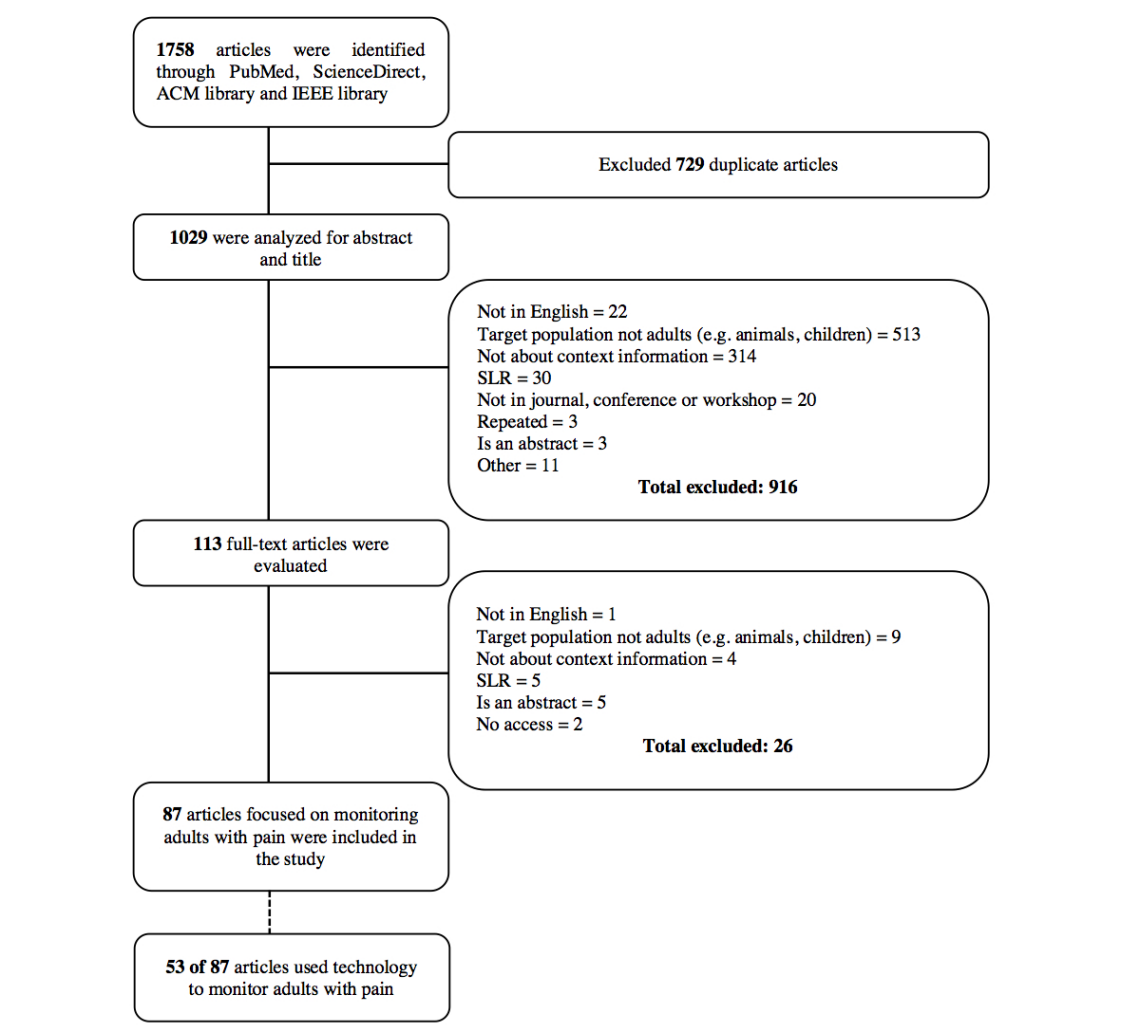
Selection flow diagram.

In total, 1758 references were identified from the databases. After removal of duplicates, 1029 publications remained. These were analyzed for abstract and title, and 911 publications were excluded because they did not meet the inclusion criteria. A total of 113 publications were evaluated for full text and 87 publications satisfied the aforementioned eligibility criteria and were included in the final review. Out of these, 53 used technology to monitor pain. The selection flow diagram for this study is presented in Figure 1.

### Characteristics of Included Studies

**Figure 2:**
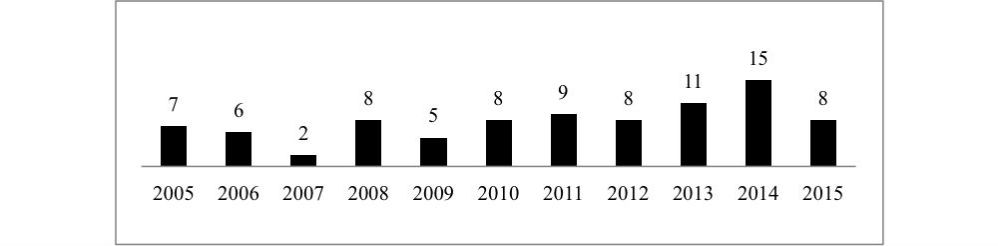
Number of publications per year.

Most of the reviewed articles were published in journals (81/87, 93%) and only 6 of 87 (7%) in conference proceedings. The distribution of studies over the years is presented in Figure 2. Of all reviewed articles, 53 of 87 (61%) presented technologies (systems, devices, apps) used to monitor adults with pain. The focus of 80% (70/87) of the research was on a specific condition, such as back pain (16/87, 18%), fibromyalgia (5/87, 6%), and neck pain (5/87, 6%). The interventions were tested on patients (57/87, 66%), on healthy volunteers (27/87, 31%), on students (2/87, 2%), or unspecified (1/87, 1%). Regarding the countries where the research was carried out, the three countries with highest representation were the United States (34/87, 39%), England (11/87, 13%), and Canada (9/87, 10%). Only 6 of 87 (7%) studies were carried out in Africa and Asia, and none in Latin America.

The selected studies collected information for several objectives. Several focused on pain relating to postures and movement in a work environment [20-29]; others studied the impact of therapy and/or exercise [30-35] and a large group saught to describe pain and the experience of pain [36-54] and pain-related pathologies [55-68]. Other studies were aimed at understanding the relationship of pain to other factors: emotional state [69-85], social context [86-88], sleep [89,90], disability [91], quality of life [92-95], and fear or catastrophism [96-98]. Some investigations proposed or evaluated technological apps for pain recognition [99], pain control [100], healthy behavior support [101], sleep monitoring [102], remote health services [103], estimating pain during therapy [104], or measuring changes after surgery [105,106].

Regarding pain measurement, 53 of 87 investigations used pain scales. From these articles, the most frequently used scale was the Numeric Pain Rating Scale (27/53, 51%), followed by the Visual Analog Scale (24/53, 45%) and Verbal Numerical Scale (2/53, 4%). Validated questionnaires were also used: McGill Pain Questionnaire (7 articles), Brief Pain Inventory (5 articles), and Multidimensional Pain Inventory (1 article); 5 articles used their own questionnaires. Six articles used two or more methods to assess pain. Only 14 articles had a control group.

### Methodology and Evaluation of the Reviewed Studies

The methodology used in each publication was analyzed. A quantitative methodology was applied in 79 of 87 studies (91%), none used only qualitative methodology, and 6 of 87 (7%) applied mixed methods. The following techniques for data collection were used: questionnaire, sensors (eg, heart rate monitor), diaries, interviews, and analysis of medical records.

The participants were asked to participate in experiments to collect data while they were monitored. The activities that participants underwent were classified into the following five categories:

1. Daily activity: monitoring the activities of a person in his/her daily life [23,27,28,60,89,90,101-103,105,106].
2. Specified activity: monitoring the activities of a person during an activity specified by the researchers, which were classified further into the following categories:
  - routine task: participants must perform a specified task (eg, reading, writing) [21,22,71,86,87];
  - physical activity: participants must engage in activities that require physical exertion (eg, lifting, cycling, walking) [20,26,29,38,51,55,59,61,64,66,91,96,97]; and
  - therapy: participants were evaluated while doing some type of therapy (eg, leg curls, music, behavioral therapy) [30-35,44,46,49,52,58,93,104].
3. Pain test: tests in which the participant feels pain (eg, hand dip tests in cold water and/or heat, or electrical stimuli) [37,39-43,45,50,53,54,70,73,81,98].
4. Display images: participants are shown images (eg, erotic, pleasant, gory images) [47,48,69,75,77-79].
5. Other: other activities [36,57,62,64,72,85,99].

**Figure 3:**
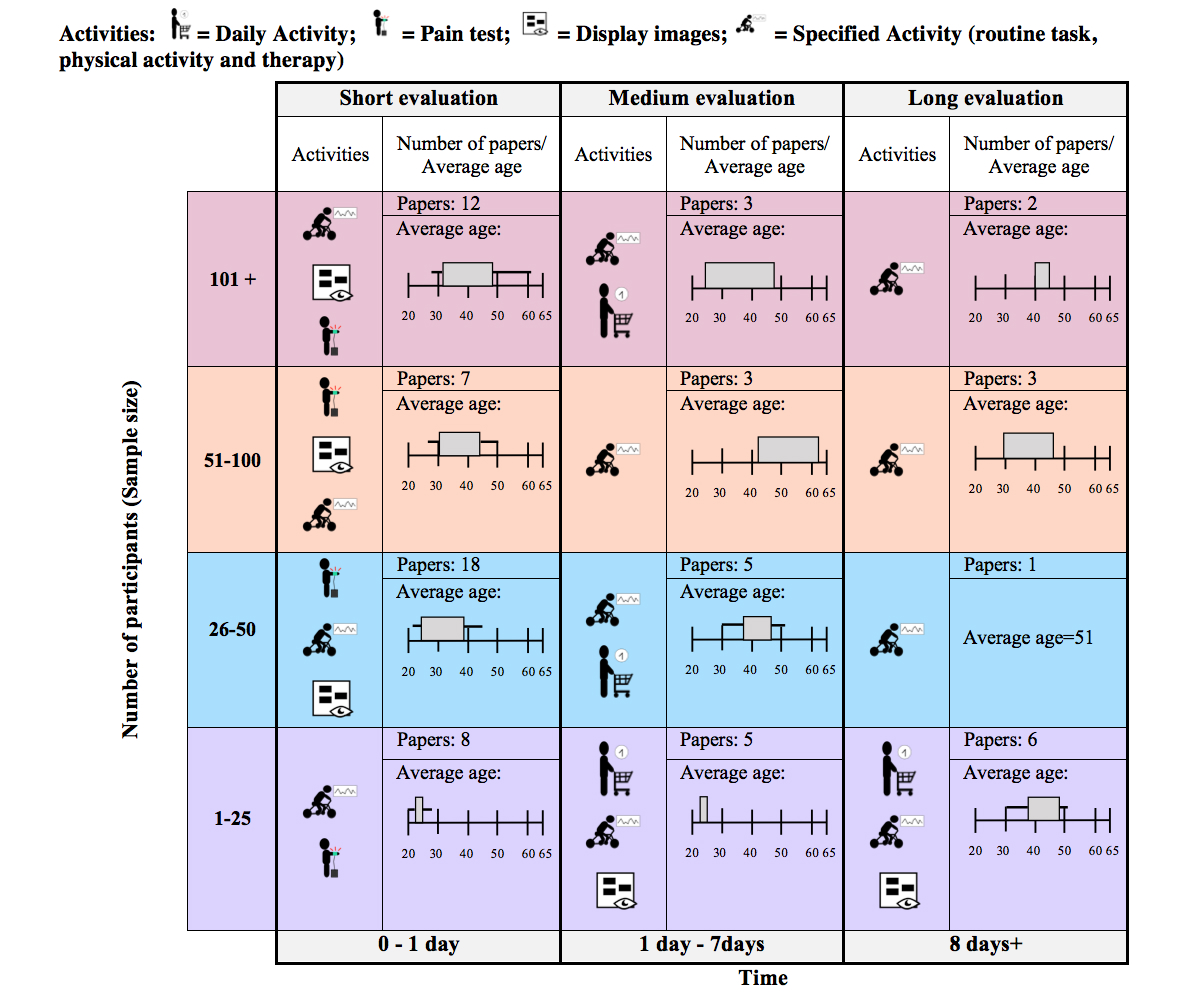
Summary of study methodologies.

The most frequent activities were pain test (14/87, 16%), therapy (13/87, 15%), physical activity (13/87, 15%), and daily activity (11/87, 13%). A summary of the methodology used in the included studies is presented in Figure 3. The studies were classified by sample size (number of participants) and duration of the evaluation. The mean age of participants in each study (when/as reported by the original research) and the activities that were included in the study (daily activity, pain test, display images, specified activity) are shown in Figure 3. Generally, studies with a longer duration used specific activities, such as therapy or physical activity, and used daily activities only when the sample size was small, possibly because daily activities are more complex to evaluate when the period of time or sample size is larger. Most of the surveyed articles had a short evaluation period, and most studies involved young people or adults, but not seniors.

### New Context Model for Pain Monitoring

Classification of the existing knowledge in a domain can provide a better understanding of the relationships between the objects, identify gaps, and ease the sharing of knowledge [1]. The 87 selected publications were reviewed and the researchers listed context information that was presented, either to study its relation to pain or to characterize pain. Then, similar context information was merged, resulting in the identification of 49 different types of contextual information.

Lienhard and Legner’s recent context model [107] included the categories activity, identity, location, and time. However, during this review, contextual information was found pertaining to new categories, and additional subcategories were found, creating 40 additional subcategories. To create the model, the context information was first categorized into one of the existing categories. Information that did not correspond to a category was placed in a separate set. The time category was eliminated because it was not collected by any of the included studies. Then, the unclassified information was grouped into sets with similar characteristics. From this analysis, three new categories were created: wellness, environment, and physiological. Finally, the location category, which did not have subcategories, was determined to be a subcategory of environment.

The 49 types of context information identified in this study are presented in Table 1, classified according to our proposed five-dimension context model. The following is a brief explanation of the categories of the model:

1. Activity: information collected from activities that require physical or mental effort by the user.
2. Identity: the user’s identifying characteristics (eg, roles, behaviors, and personality).
3. Wellness: information about a patient’s state of well-being (eg, quality of life, disability, comorbidity, and among others).
4. Environment: the patient’s surroundings (eg, noise, food, and music).
5. Physiological: data collected from the patient’s body (eg, heart rate, blood pressure, and skin conductance).

**Table 1:** New context information model for pain monitoring.

Context category and subcategory		Instruments						Articles, n		Correlation with pain, n		No correlation with pain, n	
		Questions/ interview		Medical equipment		Technical device							
**Activity**													
	Physical activity				Y		Y		7		3		1
	Mental tasks		Y				Y		1				
	Positions						Y		3		2		
	Movements						Y		4		1		
	Walk (gait)						Y		2		1		
**Identity**													
	Behavior		Y						2				1
	Personality		Y						1		1		
	Role		Y				Y		2		2		
	Ethnicity		Y						2		2		
	Coping		Y						1		1		
**Wellness**													
	Quality of life		Y						5		3		1
	Emotional state		Y						15		7		1
	Comorbidity		Y						3		1		
	Anxiety		Y						10		3		2
	Depression		Y						9		3		
	Stress		Y						12		6		
	Fatigue		Y						8		1		1
	Fear		Y						8		1		1
	Muscle injury		Y		Y				1				
	Sleep		Y				Y		7				3
	Disability		Y		Y		Y		14		1		
	Catastrophism		Y						6		2		
**Environment**													
	Vibration						Y		1		1		
	Music						Y		1		1		
	Workload		Y				Y		5		4		
	Food		Y				Y		1		1		
	Setting						Y		1				
	Voice						Y		1				
	Social support		Y						4		1		
	Noise						Y		1				
	Location						Y		3				
**Physiological**													
	Blood				Y		Y		5		1		1
	Saliva				Y		Y		3				
	Heart rate				Y		Y		15		3		2
	Blood pressure				Y		Y		8		1		
	Skin conductance				Y		Y		8		2		
	Facial expressions						Y		2		1		
	Flicker						Y		2		1		
	Pupil				Y		Y		1		1		
	Muscular strength				Y		Y		2				
	Muscle activity				Y		Y		11		2		
	Temperature				Y		Y		2				
	Column compression				Y		Y		2		1		
	Cerebral activity				Y		Y		4		2		
	Asymmetry				Y		Y		2		2		
	Musculoskeletal symptoms		Y		Y				1				
	Breathing				Y		Y		5				
	Sensitivity		Y						3		1		
	Pain tolerance		Y						4				

The context information that was collected more frequently was heart rate, disability, emotions, and depression. Most publications used physiological information (54/87, 62%) and wellness data (52/87, 60%) because these categories included heart rate, emotions, disability, and depression, which are frequently collected parameters. Next was environment information with 22% (19/87). Activity data were used in 18 of 87 publications (21%), corresponding to physical activities, mental tasks, and walking, among others. Finally was identity data with 9% (8/87).

Table 1 also presents the results of the reviewed studies, displayed next to each subcategory are the number of studies that found that the information was (or was not) correlated to pain. For example, the correlation between sleep and pain was studied three times finding no correlation, whereas stress was found by six studies to correlate to pain and other categories (eg, heart rate, fear, have conflicting results).

The instruments used to collect each type of context information are also listed in Table 1. Questionnaires or interviews were mainly used to gather subjective patient information, such as behavior, emotional state, personality, and quality of life. Medical equipment refers to specialized medical devices to collect patient information. These devices usually were handled by health professionals (eg, devices to get blood, saliva, blood pressure, and brain activity). Finally, technological devices (eg, mobile phones, mobile apps, sensors, and websites) were used to collect data through such things as online surveys and facial expression recognition.

The proposed model may be used by researchers as a base taxonomy of possible information that may be monitored in adult patients with pain; however, it is not expected that any single device should monitor all this information. Rather, researchers may select information that is relevant to their specific study, choosing to focus on information that has been found to correlate with pain or otherwise choosing to fill gaps in the literature (eg, by studying whether some of the information that is frequently monitored has a relation to pain).

### Technology to Monitor Adults with Pain

The technologies used to monitor adults with pain were studied to learn about current trends and challenges regarding pain monitoring.

#### Types of User Interfaces

A technological device includes a user interface (ie, the representation of a system with which a user can interact) [108]. There is not one agreed-on taxonomy to define every possible type of user interface, thus well-known categories of interfaces were used to classify the technologies.

##### Graphical User Interface

Graphical user interfaces represent information through an image-based representation in a display [108] and provide users with visual controls, such as menus, buttons, lists, and windows [109]. Examples of this type of interface are an electronic diary to input mood, intensity of pain, and sleep [89], electronic questionnaires [26,77,79,86,96], a mobile app [101], and laptops for sleep monitoring [102].

##### Tangible User Interface

A tangible user interface is a user interface in which a person uses a physical object to interact with digital information [110] (eg, hardware for magnetic resonance imaging) [39,70], apparatus for measuring skin conductance [39,70], joystick [87], and a motion analysis system [22].

##### Wearable User Interfaces

A wearable user interface is a device that is worn on the body (eg, embedded in clothing or accessories) [111]. This implies the use of the human body as a support environment for the devices [108]. Examples of devices that were used as wearable user interfaces in this study are mobile phones [105,106], a garment for tracking electromyography signals [101], and sensors such as accelerometers [23,60,103] and gyroscopes [66].

#### Analysis of Technologies for Monitor Adults with Pain

For each of the reviewed articles that presented monitoring technologies, the type of user interface (graphical user, tangible user, or wearable user as previously defined), the target user (either the patient him/herself or the health care worker), what type of information was monitored (according to the categories defined by our model), and the type (whether available commercially or as a research prototype) are listed in Table 2. Tangible user and wearable user interfaces were naturally used more often to collect physiological data and activity information. Physiological data were the most typically collected contextual information (27/53, 50%), whereas identity was not used, possibly because this category did not change dynamically. The devices used were overwhelmingly commercially available devices, with only four research-based devices.

The most common type of interface used to monitor adults with pain was a wearable user interface (37/53, 70%), followed by tangible user interface (22/53, 42%) and graphical user interface (9/53, 17%). Regarding wearable user interfaces, the body part where most devices were placed was the trunk (17/37, 46%) and arms (including hands; 15/37, 41%). Naturally, this was related to the type of condition that was being studied (eg, back pain was more frequently monitored through devices placed on the trunk).

The target users of these technologies were most often the patients themselves (47/53, 89%) and/or health care professionals (22/53, 42%). The devices were worn on the patients’ bodies (eg, electrodes, sensors). Most studies used these technologies not to monitor users, but rather to conduct measurements in controlled or supervised environments. Tangible user interfaces in these articles were mostly oriented toward health care professionals and not patients because they used medical equipment such as scanners or blood tests, which require special training to operate.

Using the previous classification of activities used for evaluation, the three activities that were most frequently done to evaluate technological devices to monitor adults with pain were daily activity and physical activity with 19% each (10/53), followed by pain test with 17% (9/53). Using technology allows researchers to monitor patients during their daily activities, which provides more realism and a richer context for evaluation.

### Challenges and Trends in Monitoring Adults with Pain

Five challenges in terms of monitoring adults with pain were found:

1. When monitoring is in real contexts, the user of the device must be the patient. This may generate usability challenges when users have low digital skills, as well as other technical challenges such as battery life.
2. Many contextual factors may influence pain and, as previously stated, current sensors allow measuring a large amount of information, but it is not yet clear which types of information to monitor for a particular evaluation.
3. Monitoring technology usually sends reports to health care professionals, whereas almost no feedback is given to the patients to help them understand their pain patterns, triggers, and how to adjust their activities accordingly, for example. A possible explanation for this is that medical-grade health monitors that can provide feedback to patients are rigorously tested and highly regulated [112], which results in slower adoption of new features and may lead researchers to use instead commercially available, consumer-grade monitors that do not provide feedback.
4. Most studies do not collect environmental information from the patient, although there are already sensors on the market to capture this type of information (eg, noise, humidity, temperature).
5. Increasingly, researchers have been taking advantage of available sensor technology and implementing tangible and wearable devices to monitor adults with pain in a mobile way. However, most studies did not collect data in real contexts, rather focusing on laboratory or controlled experiments.

The results were analyzed to see whether trends could be found (ie, whether changes could be detected over the time period of the review), especially regarding study methods, evaluation activities, technologies, and collected context information. No significant differences were found in the contextual data that were collected over the years nor in the types of technology used or evaluation methods.

**Table 2:** Technology used to collect context information.

Body part and user^a^				Interface^b^									Context data^c^													Type
				WUI			TUI			GUI			Wellness			Environment			Physiological data			Activity info				
**Trunk**																										
	**Patient**																									
		[105]	Y																		Y			Commercial		
		[20]	Y															Y						Commercial		
		[106]	Y																		Y			Commercial		
		[38]	Y												Y						Y			Commercial		
		[72]	Y															Y						Commercial		
		[58]	Y																		Y			Commercial		
		[59]	Y															Y						Commercial		
		[60]	Y																		Y			Commercial		
	**Patient and HCW**																									
		[33]	Y			Y												Y						Commercial		
		[23]	Y			Y									Y									Commercial/research		
		[81]	Y			Y												Y						Commercial		
		[103]	Y			Y															Y			Commercial		
		[67]	Y			Y															Y			Commercial		
		[104]	Y			Y												Y						Commercial		
		[102]	Y						Y			Y												Commercial/research		
**Arms**																										
	**Patient**																									
		[37]	Y															Y						Commercial		
		[40]	Y															Y						Commercial		
		[90]	Y																		Y			Commercial		
		[32]	Y															Y						Commercial		
		[42]	Y															Y						Commercial		
		[49]	Y															Y						Commercial		
		[78]	Y									Y												Commercial		
		[61]	Y																		Y			Commercial		
		[54]	Y															Y						Commercial		
	**Patient and HCW**																									
		[43]	Y															Y						Commercial		
		[70]	Y			Y						Y												—		
		[39]	Y			Y												Y						Commercial		
**Legs**																										
	**Patient**																									
		[55]	Y																		Y			Commercial		
		[69]	Y																		Y			Commercial		
		[66]	Y																		Y			Commercial		
**Head and neck**																										
	**Patient**																									
		[36]	Y															Y						Commercial		
	**Patient and HCW**																									
		[21]	Y			Y												Y						Commercial		
		[47]	Y			Y												Y						Commercial		
**Head, neck, and legs**																			Y							
	**Patient and HCW**																									
		[48]	Y															Y						Commercial		
**Arms and trunk**																										
	**Patient**																									
		[51]	Y															Y						Commercial		
		[101]	Y						Y												Y			Commercial		
**Arms and legs**																										
	**Patient and HCW**																									
		[77]	Y						Y			Y												Commercial		
**Not stated**																										
	**Patient**																									
		[96]							Y			Y												Commercial		
		[86]							Y			Y												Commercial		
		[89]							Y			Y												Commercial		
		[87]				Y															Y			Commercial		
		[75]				Y												Y						Commercial		
		[62]				Y												Y						Commercial		
		[26]							Y									Y						Commercial		
		[27]				Y						Y												—		
	**HCW**																									
		[22]				Y															Y			Commercial		
		[73]				Y						Y						Y						Commercial		
		[31]				Y															Y			Commercial		
		[99]				Y															Y			Commercial		
		[52]				Y												Y						Commercial		
		[63]				Y												Y						Commercial		
	**Patient and HCW**																									
		[79]				Y			Y			Y												Commercial		
		[100]				Y			Y						Y			Y						Commercial/research		

^a^ HCW: health care worker.

^b^ GUI: graphical user interface; TUI: tangible user interface; WUI: wearable user interface.

^c^ No article monitoring identity data.

## Discussion

### Principal Results

This SLR found 49 different types of context information used to monitor adults with pain and categorized them into a new five-dimensional model of context information that includes activity, identity, wellness, environmental, and physiological data. Several types of context information have been studied to see whether they correlate to pain; although publication bias tends to skew data toward positive results, we found that some contextual information has not been found to correlate to pain (eg, sleep), whereas other (eg, emotional state) has an increasing amount of evidence of its correlation to pain. This review did not find trends in the contextual information that has been presented in previous research (ie, it has not changed substantially in the 10 years of the review). Therefore, although there is potential for new sensors to allow monitoring new contextual information, there is a degree of independence between the contextual information that is of interest in the monitoring of patients with pain and sensor availability.

A total of 53 studies presented technological devices to collect context information, using wearable, tangible, and graphical user interfaces. Even though several approaches aim to capture context automatically (eg, through sensors), the proposed model makes evident that contextual information also requires manual input because patient-supplied information is relevant (eg, in the identity category of the model). Although sophisticated technologies exist for inferring emotions through facial expressions captured by video [113,114], they also need to be partly input by the patient (eg, information concerning depression, mood, anxiety), which may represent a challenge from a system usability perspective.

Recent advances in the miniaturization of biosensors, wearable technology, and microelectronics have enabled continuous ambulatory monitoring of physiological signals [115]. In this review, adults with pain were found to be more frequently monitored through wearable devices, which allow researchers to place them on the relevant body part being studied, and physiological information was the type of information most frequently captured. Wearable health monitoring technology has been found to be especially appropriate for people suffering from chronic disease, providing continuous monitoring and adequate privacy [115], and it may become pervasive for all populations due to the ubiquitousness of mobile phones and the quantified-self movement [116].

The selected articles were found to have diverse types of evaluations, spanning hours, days, or weeks, and with diverse sample sizes. The most frequent activities that participants underwent were a pain test, therapy, physical activity, and daily activity. Generally, studies with a longer duration use specific activities, whereas daily activities are used when the sample size is small, possibly because daily activities are more complex to evaluate when the period of time or sample size is larger. Usually, evaluation periods are short (less than one day), and most studies involve young people or adults, but not seniors. Only seven studies (8%) had patients older than 50 years; however, it has been found that prevalence of chronic pain does vary with age, increasing as patients age [91].

Researchers have mostly studied patient-supplied context information, and infrequently contextual information from environmental factors or patient activities. For example, studies have suggested that environmental problems may greatly affect health [117] (eg, air pollution may produce nausea [118]), but no information was found about whether this type of factor (or others such as temperature, humidity) affects pain.

### Comparison With Prior Work

Several systematic reviews related to pain have been undertaken, but they have focused on pain management (eg, therapy effectiveness [119,120] and alternative therapies [121]). This is the first work to review a large number of studies with the goal of building a model of contextual information that may be related to pain. Studies about context information and pain generally present studies in specific reduced situations (eg, a context model based on data from three interviews and for a specific solution using mobile phones [107], an ontology-based context model for patient home care for chronic diseases [122]). Likewise, research on technologies for chronic pain management only present some examples of types of technologies [123] without undertaking a structured systematic review of existing research.

### Challenges and Considerations

This study aims to provide information about contextual data that may be monitored through technological devices. Nevertheless, this area is fraught with interesting challenges. One is preserving the privacy of patients [124], especially when considering monitoring a large amount of sensitive information that may be correlated in many ways. Another is the challenge of providing adequate usability, not only in regard to interaction, but also battery life and portability. Adoption is another challenge. This requires, for example, a device to be esthetically adequate for social activities [125], and requires low amounts of interaction [126]. Designers and computer scientists will have to deal with these challenges and considerations to avoid overburdening patients and therefore negatively impacting use and adoption of monitoring devices.

### Limitations

This study only used four specific databases and only in English; therefore, more regional contributions may have been missed, which may explain our low rate of studies in Africa, Asia, and Latin America. This search was restricted to 10 years, partly to uncover recent technological proposals; however, important contextual information may have been discussed in older research articles. Also, only the word “pain” was included in the search string, omitting related words (eg, “misery” or “spasm”), which may have uncovered additional literature on this topic.

### Conclusion

A SLR was conducted with the goal of studying technologies to monitor adults with pain and relevant contextual information. Eighty-seven articles were reviewed in depth and 49 types of context information were found and organized into a five-dimension model of contextual information. Most contextual information was related to patient-supplied data and few were collected from the environment or patient’s activities. Regarding technology, wearable user interfaces are used most often to collect data and monitor patients. Nevertheless, not all information may be monitored through sensors automatically—some data must be user-supplied because some information from the patient is subjective (eg, pain intensity, fear, and emotional state).

## Abbreviations

SLR: systematic literature review
